# A do-it-yourself protocol for simple transcription activator-like effector assembly

**DOI:** 10.1186/1480-9222-15-3

**Published:** 2013-01-14

**Authors:** Claudia Uhde-Stone, Nilang Gor, Tiffany Chin, Joseph Huang, Biao Lu

**Affiliations:** 1 Department of Biological Sciences, California State University, East Bay, 25800 Carlos Bee Blvd, Hayward, CA, 94542, USA; 2 System Biosciences (SBI), 265 North Whisman Rd, Mountain View, CA, 94043, USA

**Keywords:** TALEN, TALE-TF, Golden Gate, Transcription-activator-like effector

## Abstract

**Background:**

TALEs (transcription activator-like effectors) are powerful molecules that have broad applications in genetic and epigenetic manipulations. The simple design of TALEs, coupled with high binding predictability and specificity, is bringing genome engineering power to the standard molecular laboratory. Currently, however, custom TALE assembly is either costly or limited to few research centers, due to complicated assembly protocols, long set-up time and specific training requirements.

**Results:**

We streamlined a Golden Gate-based method for custom TALE assembly. First, by providing ready-made, quality-controlled monomers, we eliminated the procedures for error-prone and time-consuming set-up. Second, we optimized the protocol toward a fast, two-day assembly of custom TALEs, based on four thermocycling reactions. Third, we increased the versatility for diverse downstream applications by providing series of vector sets to generate both TALENs (TALE nucleases) and TALE-TFs (TALE-transcription factors) under the control of different promoters. Finally, we validated our system by assembling a number of TALENs and TALE-TFs with DNA sequencing confirmation. We further demonstrated that an assembled TALE-TF was able to transactivate a luciferase reporter gene and a TALEN pair was able to cut its target.

**Conclusions:**

We established and validated a do-it-yourself system that enables individual researchers to assemble TALENs and TALE-TFs within 2 days. The simplified TALE assembly combined with multiple choices of vectors will facilitate the broad use of TALE technology.

## Background

With the recent emergence of transcription activator-like effector (TALE) technology, gene editing has entered an exciting new era [1-3]. While zinc finger nucleases have been well established for the purpose of generating targeted mutations [4], their challenging design and need for experimental optimization have restricted this technology to few, highly specialized laboratories. In contrast, TALEs are simple to design, able to target almost any DNA sequence within the genome, and promise less off-target effects compared to zinc-finger nucleases [5-7].

Native TALEs are transcription factors used by plant-pathogenic bacteria in the genus Xanthomonas. They activate transcription of host genes by binding to specific sequences in the promoter region of the targeted gene [8,9]. Strikingly, the TALE DNA binding domain consists of tandem 33–35 amino acid repeats, followed by a single half repeat of 20 amino acids. Interestingly, the tandem repeats are nearly identical, except for two amino acid codons at position 12 and 13, referred to as “repeat-variable di-residue” (RVD). Each of the four most common RVDs specifies the binding to one of the four nucleotide bases [10,11]. Taking advantage of the simplicity of the TALE coding principle, customized TALEs can be easily designed to allow genetic and epigenetic manipulation. For example, the TALE DNA-binding domains can be combined with either a catalytic DNA endonuclease domain, such as FokI, to allow gene editing, or a transcription factor (TF) domain for gene activation. Indeed, both TALEN and TALE-TF, have been successfully applied to gene-editing or activation in a number of species [2,12-16].

Because of the repetitive nature of the DNA binding domain, the assembly of customized TALEs by direct synthesis or traditional cloning is expensive and technically challenging. Realizing the potential of TALE technology, a number of approaches for TALE assembly have been devised to allow low to medium throughput ([5,17]), or high-throughput with automation [18,19]. It is worthy to note that these methods are based on the Golden Gate procedure, a cloning strategy that makes use of type IIs restriction enzymes which cut sequences adjacent, rather than within their recognition sites, and allows seamless ligation of repetitive sequences in a specific order [5,17]. The Golden Gate cloning technique has proven powerful, but typically relies on the use of large numbers of plasmids or amplified monomers, making this strategy not feasible for the individual research lab.

The objective of this research was to establish a system for simple TALE assembly, and to develop vector sets with gene editing or gene activation capability under the control of different promoters, to allow for a variety of downstream applications.

## Results and discussion

**Figure 1:**
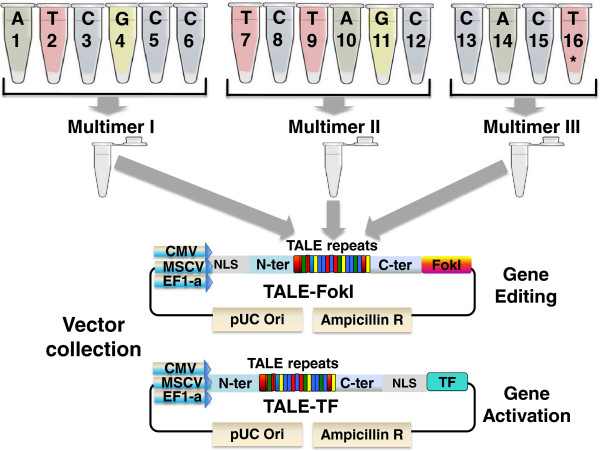
Schematic overview of multimer assembly from the ready-made monomer library into a vector of choice for gene editing or gene activation.

**Figure 2:**
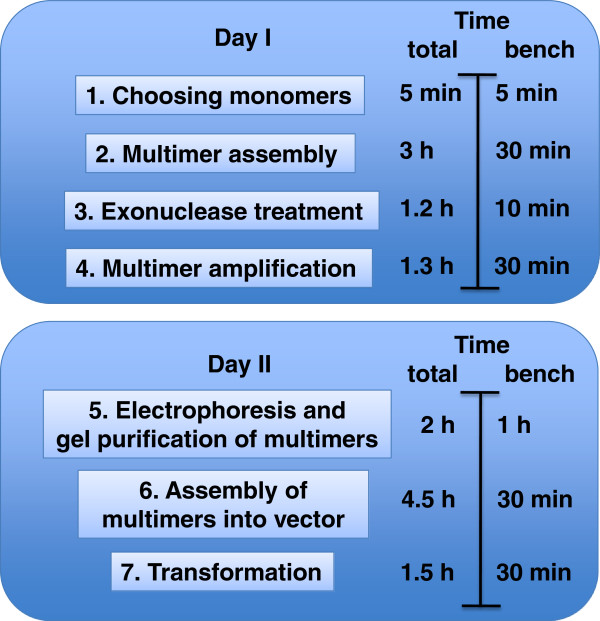
**Workflow of TALEN and TALE-TF assembly, using the do-it-yourself TALE assembly kit.** The total time and the actual hands-on time of the 7 assembly steps are indicated on the right.

We established a do-it-yourself system for the fast and simple assembly of TAL-repeats into a collection of vectors for TALEN and TALE-TF expression in mammalian cells (Figure 1). This system simplifies the assembly of custom TALEs in three main ways. First, by eliminating the need for time-consuming and error-prone set-up, it shortens time, effort and cost of assembly. Second, by providing a streamlined protocol, it simplifies an otherwise complicated approach, making it feasible for any standard molecular lab. And lastly, a collection of backbone vectors with TALEN and TALE-TF domains under the control of various promoters offers choices for diverse downstream applications in the mammalian system. Using this approach, individual researchers can assemble one or several TALEs into a vector of choice in just 2 days (Figure 2) by using standard molecular techniques and a thermocyler.

The assembly is based on the Golden Gate method, which relies on the ability of type IIS restriction enzymes to cut outside of their recognition site. Type IIS recognition sites arranged in inverse orientation at the 5' and 3' end of a DNA fragment will be removed upon cleavage, allowing simultaneous restriction and ligation. The continuous re-digestion of unwanted ligation products increases the formation of the desired construct. As type IIS fusion sites can be designed to have different sequences, Golden Gate cloning enables directional and seamless assembly of multiple DNA fragments.

**Figure 3:**
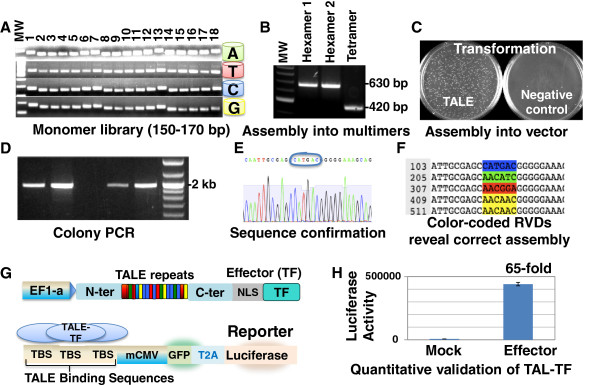
**A) A ready-made library of normalized, quality-controlled monomers provides the building blocks for TALE assembly. B)** According to the custom TALE design, monomers are assembled into 2–3 multimers in a restriction and ligation-based procedure, using a thermocycler. In the example shown here, multimer 1 and 2 are hexamers while multimer 3 is a tetramer. **C**) *E. coli* transformation with the final assembly product in a vector of choice typically results in tens to hundreds of colonies, while the negative control should have significantly lower to no colonies **D)** Correct assembly of multimers into the vector can be assessed by colony PCR, and further confirmed by sequencing **(E)**. **F)** As the TALE binding specificity is based on 4 types of RVDs, color-coding the RVD-encoding nucleotides can quickly reveal the correct order of tandem repeats. **G**) For functional validation, a custom TALE domain was assembled into the EF1-TALE-TF vector. In addition, a dual reporter construct was generated carrying 3 copies of the TALE binding sequence. **H)** Co-transfection of the custom TALE-TF and the dual reporter construct into HEK293 cells showed strong induction of luciferase activity, confirming the TALE-TF functionality.

As a first step of our do-it-yourself protocol, we assembled monomers into multimers (Figure 3A, B), using a procedure based on restriction, ligation and amplification. Multimer 1 and 2 are designed to be hexamers, but the length of multimer 3 can vary to allow variations in the final length, such as 14–19 bp binding sequences. To remove the incompletely assembled and thus linear ligation products, DNA exonuclease treatment was carried out after the multimer assembly. The correctly assembled circular multimers were subsequently amplified by PCR.

On day 2, gel-purified multimers were assembled into a vector of choice, using a second restriction-ligation-based procedure, followed by bacterial transformation (Figure 3C). Colony PCR was performed for confirmation of insert size (Figure 3D); typically, 40-90% of colonies displayed correct insert size. We recommend to use two colonies of correct insert size for sequence confirmation (Figure 3E, F); typically 80-90% of sequences revealed correct assembly. A detailed protocol of this approach is provided in the Materials and Methods section.

We have successfully assembled a number of custom TALEs into various vectors. To examine functionality of an assembled TALE, we performed a co-transfection experiment in human embryonic kidney 293 cells. Consistent with previous findings [20], the custom-assembled TALE-TF activated luciferase activity 65-fold, validating its functionality (Figure 3G, H).

**Figure 4:**
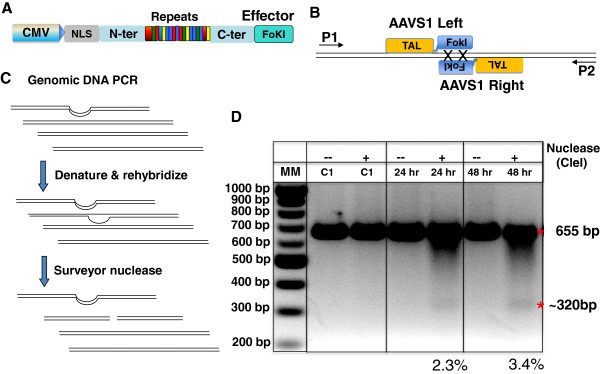
**TALEN activity in HEK293 cells. A**) This schematic shows the general configuration of TALENs that were assembled under the control of a CMV promoter. **B**) The nucleotide sequences of target sites are 5-TGCCCCTCCACCCCCAC-3’ (top strand), and 5’-TTTCTGTCACCAATCCTG-3’ (bottom stand) flanking a 16-bp spacer. The primers for genomic PCR are 5’-CTTGCTTTCTTTGCCTGGAC-3’ (P1) and 5’-GATCAGTGAAACGCACCAGA-3’ (P2). **C**) Schematic of the Surveyor nuclease mutation detection assay used to determine TALEN *de novo* cleavage. **D**) 2% agarose gel showing the Surveyor nuclease mutation detection result from the assembled AAVS1 TALEN pair. Controls (C1) from mock-transfected HEK293 cells showed no cutting with Surveyor nucleases, whereas cells transfected with the AAVS1 TALEN pair produced expected ~320 bp products. The efficiency was 2.3% and 3.4% at the 24-hour and 48-hour time-points, respectively.

To confirm functionality of an assembled TALEN, we performed a transfection experiment in human embryonic kidney 293 cells using a pair of assembled TALENs to target the AAVS1 locus in the human genome (Figure 4A). For TALEN activity, the Surveyor nuclease mutation detection assay provides a functional validation of successful *de novo* cutting with a particular pair of TALENs. As shown in Figure 4B, we designed primers that have an amplicon of 650 bp and flanking the centrally localized TALEN target. PCR-amplifcation using this primer pair produced a single band in both mock- and TALEN-transfected samples. After denaturing, heteroduplex reannealing and Surveyor nuclease treatment, AAVS1 TALEN-transfected cells displayed an extra band of ~320 bp as predicted (Figure 4C-D). These data validate the function of assembled TALENs using Surveyor nuclease mutation detection assay.

## Conclusions

We established and validated a do-it-yourself strategy that enables researchers to assemble TALE-TF/TALENs in just 2 days. The simplicity of this approach and its minimal hands-on time makes gene-editing an affordable and practical choice for the standard molecular lab. The choices of a number of useful vector sets should further broaden TALE technology to various applications.

## Methods

### Experimental design

Free online tools such as TALEN Targeter [6] and idTALE [21] are available to design TALENs and TALE-TFs that are specific and have a low risk of off-target effects. TALE-TFs only require one effector protein, while TALENs require the design of protein pairs, which bind two DNA sites, usually spaced 15–30 bp apart to allow for optimal FokI dimerization and cutting [5]. Length of DNA binding sequences may vary, typically ranging from 14–20 bp. In humans, 20 bp may offer high specificity, considering the genome size. It is worthy to note that longer domains (18–20 bp) may decrease cell toxicity by reducing the risk of off-target effects [19].

The appropriate vector can be chosen from a collection of TALEN and TALE-TF backbone vectors, listed in Table 1, according to the desired downstream application. As the last nucleotide of the DNA target is vector encoded, all vectors come in 4 flavors, containing an A, T, G, or C-specific half repeat.

**Table 1:** Available TALE backbone vectors

**Vector^1^ name**	**Promoter**	**Application**
CMV-TALEN-NI	Cytomegalovirus (CMV)	Targeted deletions
CMV-TALEN-NG		
CMV-TALEN-HD		
CMV-TALEN-NN		
EF1-TALEN-NI	Elongation Factor 1-alpha (EF1)	
EF1-TALEN-NG		
EF1-TALEN-HD		
EF1-TALEN-NN		
MSCV-TALEN-NI	Murine Stem Cell Virus (MSCV)	
MSCV-TALEN-NG		
MSCV-TALEN-HD		
MSCV-TALEN-NN		
CMV-TALE-TF-NI	Cytomegalovirus (CMV)	Targeted transcriptional activation
CMV-TALE-TF-NG		
CMV-TALE-TF-HD		
CMV-TALE-TF-NN		
EF1-TALE-TF-NI	Elongation Factor 1-alpha (EF1)	
EF1-TALE-TF-NG		
EF1-TALE-TF-HD^2^		
EF1-TALE-TF-NN		
MSCV-TALE-TF-NI	Murine Stem Cell Virus (MSCV)	
MSCV-TALE-TF-NG		
MSCV-TALE-TF-HD		
MSCV-TALE-TF-NN		

^1^ All vectors come as a set of four, with RVDs NI (A), NG (T), HD (C) or NN (G) specifying the nucleotide binding of the terminal half repeat. ^2^Note that the half-repeat RVD in the EF1-TALE-TF-NG vector is encoded by AAT GGC instead of AAC GGA, and that of the TALE-TF-NN vector is encoded by AAT AAC instead of AACAAC.

### Materials

#### Reagents

EZ-TAL™ assembly kit (System Biosciences, Mountain View, CA), includes monomer library, Combo Buffer I, Combo Buffer II, *Bsm*BI restriction enzyme , *BSa*I restriction enzyme, T7 Ligase, Exo buffer, Exonuclease, High-fidelity PCR buffer, High-fidelity DNA polymerase, BSA, DTT, ATP, dNTP, Multimer primer mix, Colony-PCR primer mix, Sequencing primer SF-1 (forward-1), Sequencing primer SF-2 (forward-2), and Sequencing primer SR (reverse).

Additional Reagents not provided in EZ-TAL™ base kit

EZ-TAL™ end monomers for target size 14–19 (System Biosciences)

Taq polymerase (New England BioLabs, Ipswich, MA, #MO267X)

Ultra-Pure Agarose (Life Technologies, Grand Island, NY, #15510-027)

Agarose Gel Loading Dye Blue (6x) (New England BioLabs, #B70215)

Quick-Load 2-log DNA Ladder (New England BioLabs, #N04695)

1X TAE Electrophoresis Buffer (40 mM Tris, 20 mM acetic acid, and 1 mM EDTA).

Ethidium Bromide (Amresco, Solon, OH, #X328)

QIAquick Extraction Kit (Qiagen, Valencia, CA, #28706)

Chemically Competent *Escherichia coli,* such as One Shot Stbl3 chemically competent *E. coli* (Life Technologies # C7373-03)

SOC medium (Life Technologies, #46-0700)

LB-Carbenicillin Plates (Teknova, Hollister, CA, #LI008)

LB Broth, (Teknova, #L8000)

Carbenicillin (Teknova, #C2130)

### Equipment

Thermocycler (PTC-2000 Peltier Thermal Cycler, MJ Research)

Microfuge tubes (Eppendorf, Hauppauge, NY, 022363204)

Flat-cap PCR tubes (Bio-Rad, Hercules, CA, #TFI0201)

Micropipetters and tips (Rainin, Columbus, OH)

Gel electrophoresis equipment for agarose gels (Bio-Rad)

Gel documentation system (ChemiDOC, Bio-Rad)

Incubator for Heat Shock Treatment (Thermomixer R, Eppendorf)

37°C Shaker Model G25 Incubator/Shaker, New Brunswick Scientific)

Culture test tubes with cap (17x100 mm), (Fisherbrand, Houston, TX, #14-956-6B)

## Protocol

### TALE assembly

#### Day 1

1. Choosing monomers (5 min).

Divide the target sequences of 14-20 nucleotides into multimers, excluding the first (5’) T and the last (3’) nucleotide, which is vector encoded. The first two multimers should be hexamers, the last multimer is variable in size and contains however many monomers remain (excluding the vector-encoded last nucleotide).

Take the corresponding color-coded monomers from the EZ-TAL™ kit (Figure 1). A special set of “end” monomers can be used to adjust for target sizes shorter than 20 nucleotides (14-19).

Example T | A T C G C C | T C T A G C | C A C T* | G

2. Multimer assembly (bench time 30 min, total time 3 hours).

In a separate tube for each multimer, pipette 1 μL of each monomer; adjust last multimer to 6 μL with H_2_O if necessary.

Combine components specified in Table 2. Add 4 μL of mix to each multimer for a total of 10 μL. Place each multimer tube in a thermocycler and use cycling conditions specified in Table 3 for ~ 2.5 hours.

**Table 2:** Components for multimer assembly

**Component**	**Amount per 1 reaction (μL)**
Combo Buffer	1
DTT	1
ATP	1
*Bsm*BI restriction enzyme	0.75
T7 Ligase	0.25
total	4

**Table 3:** Thermocycle conditions for multimer assembly

**Cycle Number**	**Temperature 1**	**Temperature 2**
1	37°C, 5 min	
2-16	20°C, 5 min	37°C, 5 min
Hold at 4°C		

Example

Multimer 1: A1 + T2 + C3 + G4 + C5 + C6

Multimer 2: T7 + C8 + T9 + A10 + G11 + C12

Multimer 3: C13 + A14 + C15 + T16^end^ + 2 μL H_2_O

3. Exonuclease treatment (bench time 10 min, total time 1.2 hours)

To degrade any noncircular ligation products, add components specified in Table 4.

**Table 4:** Components for exonuclease treatment

**Component**	**Amount per 1 reaction (μL)**
Exo buffer	1.5
ATP	2
Exonuclease	1.5
total	5

Add 5 μL of master mix to each multimer tube, for a total of 15 μL. Place each tube in a thermocycler and use cycling conditions specified in Table 5 for 1 hour.

**Table 5:** Thermocycle conditions for exonuclease treatment

**Cycle Number**	**Temperature 1**	**Temperature 2**
1	37°C, 30 min	70°C, 30 min
Hold at 4°C		

4. Multimer amplification (bench time 30 min, total time 1.3 hours)

To amplify each multimer, combine components specified in Table 6. Add 49 μL of mix to 1 μL of multimer template each. Perform multimer PCR using conditions specified in Table 7.

**Table 6:** Components for multimer amplification

**Component**	**Amount per 1 reaction (μL)**
PCR buffer	10
DNA-grade Water	35.5
Multimer primer mix	2.5
dNTP	0.5
High-fidelity DNA polymerase	0.5
total	49

**Table 7:** Multimer PCR conditions

**Cycle Number**	**Denature**	**Anneal**	**Extend**
1	95°C, 2 min		
2-36	95°C, 20 s	60°C, 20 s	72°C, 30 s
37			72°C, 3 min
Hold at 4°C			

#### ***Day 2***

5. Gel electrophoresis and purification of multimers (bench time 1 hour, total time 2 hours)

Run all 50 μL sample of each amplified multimer in 1 large well or 2 medium-sized wells on a 2% agarose gel; include a molecular weight marker. Excise multimer bands of correct size. To estimate the correct band size, multiply the number of assembled monomers by 103 bp and add 20 bp. For example, a hexamer will run at about 640 bp. When excising the bands, avoid cross-contamination between multimers that are intended for assembly of different TALEs.

While bright multimer bands in the range of 20 ng/μL and above are preferred, lower concentrations (10 ng/μL range) can be assembled, though with lower efficiency. Purify the bands using an appropriate gel purification kit, such as QIAquick Extraction Kit (Qiagen, Valencia, CA) following manufacturer’s instructions.

6. Assembly of multimers into vector (bench time 30 min, total time 4.5 hours)

Choose an EZ-TAL™ backbone vector according to the intended downstream application (see Table 1 for a list of available backbone vectors). The vector should contain the half-repeat that specifies the last nucleotide of the target DNA. For example, if the DNA target ends with T, use the T-version of the vector.

Combine the corresponding multimers, vector, and components of the EZ-TAL™ kit as specified in Table 8 in a PCR tube for a total of 10 μL. Include a negative control as shown in Table 8. If concentration of multimers is below the recommended range (below 20 ng/μL), reduce vector concentration accordingly. Place tubes in a thermocycler and use the cycling conditions specified in Table 9.

**Table 8:** Components for assembly of multimer into vector

**Component**	**Amount per 1 reaction (μL)**	**Negative Control (μL)**
Backbone Vector	1	1
3 Purified Multimers	5*	0
ATP	1	1
T7 Ligase	0.25	0.25
Combo Buffer II	1	1
*BSa*I enzyme	0.75	0.75
BSA	1	1
DNA-grade H_2_O	0	5
Total	10	10

*The proportion of each multimer within these 5 μL can be adjusted to achieve a similar concentration of each multimer in the mix.

**Table 9:** Thermocycle conditions for assembly of multimers into vector

**Cycle Number**	**Temperature 1**	**Temperature 2**	**Temperature 3**
1	37°C, 5 min		
2-20	20°C, 5 min	37°C, 5 min	
21			80°C, 20 min
Hold at 4°C			

7. Transformation (bench time 30 min, total time 1.5 hours)

Transform competent *E. coli* with the assembly product, following manufacturer’s instructions. Plate transformed *E. coli* on LB with Carbenicillin (100 μg/L); alternatively, Ampicillin (100 μg/L) can be used. It is expected to see tens to hundreds of colonies on plates transformed with the assembled product, while the negative control plates should display significantly fewer or no colonies (Figure 3C).

### TALE assembly confirmation

#### *Colony PCR* (bench time 30 min, total time 2.2 hours)

Perform a colony PCR of 5–10 colonies per TALE assembly. For template, slightly dip a sterile pipette tip into a colony, streak an LB plate once to keep track of the colony, and then dip the pipette tip into a tube with 100 μL sterile H_2_O. For negative control, swipe over agar plate between colonies, and process like a colony dip. Label colony streaks according to tube numbers, and incubate over night at 37°C.

Set up a colony PCR as specified in Table 10. Add 19 μl of mix to 1 μL of diluted template (colony suspension or negative control in 100 μL H_2_O) for a total of 20 μL. Place tubes in thermocycler, and run using cycling conditions as specified in Table 11.

**Table 10:** Components for colony PCR

**Component**	**Amount (μL)**
dNTP	0.2
Colony-PCR primer mix	0.2
Taq Polymerase (5 U μL^-1^)	0.1
Taq Polymerase buffer, 10x	2
DNA-grade H_2_O	16.5
total	19

**Table 11:** Thermocycle conditions for colony PCR

**Cycle Number**	**Denature**	**Anneal**	**Extend**
1	94°C, 3 min.		
2-31	94°C, 30 s	60°C, 30s	72°C, 2 min
32			72°C, 5 min
Hold at 4°C			

#### *Agarose gel electrophoresis of colony PCR* (bench time 30 min, total time 1.5 hours)

Run all 20 μL of the colony-PCR samples and negative control on a 1% agarose gel; include a molecular weight marker. The expected size of the band can be calculated as number of inserted monomers x 103 bp plus 250 bp.

#### Sequence confirmation

Inoculate 2 colonies that show a single band of correct size in LB + Carbenicillin (100 μg/L) or Ampicillin (100 μg/L), and incubate over night for plasmid isolation. For sequence confirmation, use sequencing primers provided in the EZ-TAL™ kit, EZ-TAL™ SF1 (forward-1), EZ-TAL™ SF2 (forward 2), and EZ-TAL™ SR (reverse). For short TALEs with DNA target sites of 14–15 bp, primer EZ-TAL™ SF2 can be omitted.

## Trouble-shooting advice

### Poor multimer amplification

A faint or missing multimer band in combination with smear in the high molecular-weight range may indicate failed or impaired exonuclease treatment. Ensure correct storage conditions of exonuclease enzyme and ATP, and make sure that all components are added in correct amounts. Do not skip or shorten exonuclease incubation time.

### Colony PCR does not show bands of correct size

To assess quality and quantity of the gel-purified multimers, run an aliquot on an agarose gel. Multimers of 10 ng/μL and less can still be assembled, but with reduced efficiency. If concentration of multimers is lower than recommended (below 20 ng/μL), reduce vector concentration in the assembly step (step 6) accordingly. If necessary, redo any multimer of low concentration and/or quality.

### Sequence error

About 10-20% of sequenced clones may display a sequence error, such as an incorrect repeat or a frame shift. In this case, we recommend sequencing some additional clones. If this does not reveal an error-free sequence, we recommend to redo the multimer that contains the error, and re-assemble into the vector. Note that the half-repeat RVD in the EF1-TALE-TF-NG vector is encoded by AAT GGC instead of AAC GGA, and that of the TALE-TF-NN vector is encoded by AAT AAC instead of AACAAC (see Table 1, footnote).

## Abbreviations

C-ter: Carboxy terminus; CMV: Cytomegalovirus promoter; EF1-α: Elongation Factor 1-alpha promoter; GFP: Green Fluorescent Protein; mCMV: minimal CMV promoter; MSCV: Murine Stem Cell Virus promoter; MW: Molecular Weight Marker; NLS: Nuclear localization signal; N-ter: Amino terminus; ORI: origin of replication; R: Resistance; RVD: repeat-variable di-residue; TALE: Transcription activator-like effector; TALEN: Transcription activator-like effector nuclease; TF: transcription factor; T2A: Self-cleaving 2A peptide sequence.

## Competing interests

BL, NG, TC and JH declare financial competing interest as SBI (System Biosciences) employees. CUS declares competing interest as a collaborator with SBI.

## Authors’ contributions

CUS and BL conceived the study aims and design. CUS, BL, NG and TC performed the experiments and interpreted the results. CUS drafted the manuscript. BL, NG, TC and JH contributed to the revision of the manuscript. All authors have read and approved the final version of this paper.
